# From virtual reality to augmented reality: A neuromarketing perspective

**DOI:** 10.3389/fpsyg.2022.965499

**Published:** 2022-09-09

**Authors:** Vincenzo Russo, Marco Bilucaglia, Margherita Zito

**Affiliations:** ^1^Department of Business, Law, Economics and Consumer Behaviour “Carlo A. Ricciardi”, Università IULM, Milan, Italy; ^2^Behavior and Brain Lab IULM, Neuromarketing Research Center, Università IULM, Milan, Italy

**Keywords:** augmented reality, virtual reality, neuromarketing, consumer neuroscience, consumer behavior

## Neuromarketing, virtual reality, and augmented reality in consumer behavior

Recent studies on consumer behaviors showed that the role of neuroscientific tools in increasing the knowledge of the neural mechanism is involved in the decision and emotional processes (Russo et al., 2022). Neuromarketing refers to the use of such tools in business practices, especially in advertising and marketing research (Ramsøy, 2019). It aims to overcome the limitations of the traditional methodologies by directly investigating emotional and cognitive reactions through electrophysiological and biometric measures (Karmarkar and Plassmann, 2019). In fact, traditional research uses self-report measures to investigate emotions, but this measurement is not sufficient to capture the complexity of the emotional experience since it is based on cognitive processes allowing to only investigate the conscious side of emotions (Micu and Plummer, 2010), and it is subjected to cognitive bias or social desirability (Missaglia et al., 2017). Neuroscience applied to consumer psychology can be crucial in understanding the role of advertising or other stimuli in the consumers’ processing phases, wherein emotions can build meaning (Passyn and Sujan, 2006).

Nowadays, consumers’ needs are not only related to the product/service as such but also to the associated buying experience that must be enchanting, captivating, and fascinating (Kazmi et al., 2021). Virtual Reality (VR) and Augmented Reality (AR) technologies represent suitable candidates, given their abilities to produce intense and enhanced experiences (Slater et al., 2020). In marketing, VR has been investigated in different areas, such as the product and offer perception (Grudzewski et al., 2018), the impact on B2B buyer perceptions (Boyd and Koles, 2019), the evaluation of brands, and how they can be perceived depending on the vividness effect of VR (Van Kerrebroeck et al., 2017), and the tourism sector with highlights for the tourism promotion (Adachi et al., 2020). In marketing, AR is considered “a strategic concept that integrates digital information or objects into the subject’s perception of the physical world, often in combination with other media, to expose, articulate, or demonstrate consumer benefits to achieve organizational goals” (Rauschnabel et al., 2019, p.44). Thanks to 3D virtual objects or environments -most of which are interactive (Javornik, 2016a)–placed into the real scene, it enhances the user experience (Rauschnabel et al., 2022) and creates new ways to deliver information and show products (Huang and Liao, 2015).

In defining AR, scholars argue that it is somehow related to VR because of its immersivity: in both cases, users feel telepresence, namely, the sensation to be present in an environment that is real, with the possibility to respond, and control and act on the experience they are living (Steffen et al., 2019). VR scenarios are separated from the person (Preece et al., 2015), computer-generated, and depleted of elements belonging to the physical environment (Javornik, 2016a), while AR combines real objects with virtual elements (Rauschnabel et al., 2018, 2022) that match the physical environment in terms of real-time perception and interactivity (Javornik, 2016b; Rauschnabel et al., 2022). Beyond interactivity, which produces higher engagement (Esteban-Millat et al., 2014) and influences consumer responses (Gao et al., 2009), augmentation represents a key element in the AR since it redraws the physical reality (Preece et al., 2015). In the marketing field, augmentation applies to the person, the space, and the product. The augmentation of the person is applied in virtual fitting rooms, belonging to the field of the so-called virtual try-on. Also known as a virtual or magic mirror (Javornik et al., 2016), this type of augmentation is particularly used by cosmetic brands (Javornik, 2016a). The augmentation of the space can be found in the product placement or interior design areas, where virtual products can be placed in a real environment, giving consumers the idea of that placement (Jessen et al., 2020). Finally, the augmentation of products, based on interactive elements, provides additional information about real objects whose logo or QR code is scanned using smartphone apps (Do et al., 2020).

Even though VR and AR are similarly evolving in both the theoretical models and applicative examples (Loureiro et al., 2020), they still differ in popularity among consumer behavior researchers: only a few studies focused on AR (Javornik, 2016b; Kazmi et al., 2021), in contrast with the VR. Thus, focusing on AR emerged as crucial. This technology can be used to verify behavioral models related to the decision-making and the consumers’ approach/avoidance experience (Loureiro et al., 2020), as well as to test predictive models for the consumer responses (Sundar et al., 2015). As technology augments the users’ experience, it better supports their needs, influencing the whole experience and enhancing emotions linked to pleasure and arousal (Kourouthanassis et al., 2015). This would be functional to better capture the users’ experience: understanding emotions, particularly in the precise moment in which they are felt, allowing them to understand the experimentation of a stimulus and the possibility that a message can influence the individuals’ behavior (Donovan and Henley, 2003). Moreover, the technology usage has psychological implications since its interactivity can give a sense of control, or the personalization can flow into a sense of agency (Sundar et al., 2015), also boosting the brand image and the willingness to repeat the experience (Obada, 2013; Esteban-Millat et al., 2014).

Considering these elements, we argue the importance of deepening the role of AR in the emotional response of consumers to a product and to an experience that is closer to reality. Together with the mentioned engagement the combination of real objects with virtual elements matching the physical environment (Javornik, 2016b; Rauschnabel et al., 2022) and redrawing the physical reality (Preece et al., 2015), AR can be a precious element to investigate real-time consumers’ experiences with neuromarketing technique. With regards to neuromarketing, although the context of VR has already been explored, AR neuromarketing is still underrepresented. Supplying this scarcity in the literature is important, as AR technology is applied more and more in different marketing fields. One example is in retail, where AR gives the augmentation of a product or a person (Rejeb et al., 2021), and beauty and cosmetics, where the use of magic mirrors provides recommendations for the makeup (Almeida et al., 2015), as well as the visual merchandising area, where augmented objects can be positioned in a real store (Jessen et al., 2020). In the e-commerce field, friendly shopping cart software can be used to boost the buying experience (Yaoyuneyong et al., 2014), similar to tourism, with tools for improving the immersion experience based on the augmentation of objects and environments, such as hotels rooms, restaurants, and museal contents (Siamionava et al., 2018). Finally, also healthcare marketing is adopting AR during the product evaluation (Renu, 2021).

Considering the characteristics of the associated media (Javornik, 2016a; Do et al., 2020), AR can be studied since they play a key role in measuring the impact of technology to the consumers, as well as in evaluating the interactions between human and technology (Li and Meshkova, 2013; Sundar et al., 2015). As argued by these studies, if at the beginning, AR was expensive and not fully accessible, now, technological development has cut the costs and made the use of AR within different devices possible. It can be embedded into fixed devices such as screens or mirrors, as well into mobile devices such as smartphones or wearable devices such as smart glasses (Yaoyuneyong et al., 2016; Rauschnabel et al., 2018, 2019). By creating an experience more immersive, enjoyable, and useful, AR technology can rise positive attitudes toward the used media, as shown, for instance, in the e-commerce field (Esteban-Millat et al., 2014; Yim et al., 2017).

## Neuromarketing tools for virtual reality and augmented reality experience

As mentioned, the VR experience has already been investigated through neuromarketing techniques. A study by Uhm et al. (2019) suggested that subjects experimenting with a high sense of presence in a virtual environment show high arousal; a study by Ayata et al. (2017) tried to understand the consumers’ behavior in a virtual supermarket; whereas a study by Ostrovskaya and Vives (2020) highlighted the importance of neuromarketing tools to capture users’ preference while booking hotels rooms *via* VR experience. Considering the possibility of highly simulating the real environment through AR technology, it is important to also focus on neuromarketing tools to capture emotional responses in AR situations. This is particularly important as little has been done in AR with neuromarketing techniques, also with the understanding of the AR features in consumer behavior (Gill and Singh, 2022). As for the specific tools that were suggested to deepen research, the electromagnetic activity of the brain can be measured by electroencephalography (EEG) to assess, on a moment-to-moment basis, the brain changes related to memory activation, interest, or engagement (Zito et al., 2021a). Some tools record non-brain activity, such as eye tracking, which is mainly used to assess visual salience and exploration patterns, giving indications of attention (Russo et al., 2021). In addition, by measuring the pupil diameter, the eye-tracker provides information on emotional arousal and cognitive workload (Bilucaglia et al., 2019). Skin conductance is used to detect physiological activation: when arousal occurs, an increase in sweat secretion dealing with an increase in skin conductance is observed (Zito et al., 2021b). Heart rate, which reflects both the sympathetic and parasympathetic branches of the autonomous nervous system, can predict various cognitive and emotional states, such as engagement, attention, and emotional valence (Barraza et al., 2015). Finally, facial coding is used to measure, through human expressions, the experimented feelings. Based on Eckman’s studies, this technique can detect the emotional impact of stimuli by the evaluation of unobservable micro-muscle changes (Stasi et al., 2018).

It is important to consider the nature of AR and the exposure to specific visual stimuli that could engage but also ask for an effort of adaptation that can change the human’s sensitivity and sensorial perception (Baldassi et al., 2018). Therefore, neuromarketing emerges as an important element in the consumer responses to AR investigation. Neuromarketing studies could benefit from the AR technology as a new way to deliver marketing stimuli to investigate the emotional and cognitive impacts of augmented products, such as augmented food packages or augmented brand logos. In addition to different buying behaviors (e.g., perceived value, likelihood to purchase), novel eye-tracking patterns, as well as memorization, attention, and engagement values, are expected.

Neuromarketing tools have been already used within the immersive VR technology through devices applied to the head and the hands to record consumers’ responses and changes in muscle control over a long time. This was possible since VR involves the sensorimotor system more than other stimuli, with the potential of increased realistic psychological and behavioral responses (Bohil et al., 2011). Recent studies in the Brain-Computer Interfaces tested the EEG technology in the VR field, with the aim to produce a more immersive experience for the subjects, through highly interactive and imaginative situations. This procedure is also to be applied to AR technology through applications that are able to give biofeedback from brain and body signals (Cardin et al., 2016; Loureiro et al., 2020).

## Discussion

This article aimed to focus on the importance of deepening the role of AR in the emotional response of consumers to a product and an experience through specific tools, such as neuromarketing techniques, considering that AR could be closer to reality than other simulated situations. If VR has already been investigated, AR should be deepened by scholars, researchers, and companies for them to offer reliable answers on consumer behavior in augmented situations.

These considerations can be useful in tracing a roadmap for future research in the neuromarketing and AR fields. Neuromarketing studies could include AR as an alternative experimental environment, while AR could consider the benefits of the neuromarketing tools (Alimardani and Kaba, 2021).

Augmented Reality technologies will directly mediate the interaction between the brain and the real world, as well as the effort in adaptation and perception of risks, including cognition, memory, and decision-making (Baldassi et al., 2018). In this sense, neuromarketing tools could prevent these risks by monitoring brain and physiological signals, which can undermine the consumers’ health and decision-making process. AR has the potential to create a world similar to reality, in which consumers can experiment with objects, self-augmentation, and different spaces, allowing brands to show how to use, enjoy, and engage with products. This is also functional to fully understand consumers’ responses, through the measure of their emotions. To do this, understanding what influences the interaction between consumers and technology is crucial. This should consider both the possibility to control these responses through biofeedback detection and the usage and users’ emotional response to a specific media applied to AR (o VR) technology. This is functional and essential to plan and combine AR technology with the right product and media to offer a real and enjoyable experience that consumers will repeat, dealing with consumers’ needs to associate an experience with the product.

## Author contributions

VR, MB, and MZ wrote the first draft and each section of the manuscript, contributed to the manuscript’s final writing and revision, read, and approved the submitted version. All authors contributed to the article and approved the submitted version.
